# Impacts of Hydrothermal Treatments on the Morphology, Structural Characteristics, and In Vitro Digestibility of Water Caltrop Starch

**DOI:** 10.3390/molecules26164974

**Published:** 2021-08-17

**Authors:** Jia-Lin Liu, Po-Ching Tsai, Lih-Shiuh Lai

**Affiliations:** Department of Food Science and Biotechnology, National Chung Hsing University, 145 Xingda Road, Taichung 40227, Taiwan; a0979629266@gmail.com (J.-L.L.); ss40616ss@gmail.com (P.-C.T.)

**Keywords:** hydrothermal treatment, pasting characteristics, structural properties, digestibility

## Abstract

The influence of hydrothermal treatments on the structural properties and digestibility of water caltrop starch was investigated. Scanning electron microscopy (SEM) showed some small dents on the surface of starch granules for samples treated with heat moisture treatment (HMT), but not for samples treated with annealing (ANN) which generally showed smoother surfaces. The gelatinization temperature of starch was generally increased by hydrothermal treatments, accompanied by a trend of decreasing breakdown viscosity. These results implied the improvement of thermal and shearing stability, particularly for HMT in comparison to ANN. After being cooked, the native and ANN-modified water caltrop starch granules were essentially burst or destroyed. On the other hand, the margin of starch granules modified by HMT and dual hydrothermal treatments remained clear with some channels inside the starch granules. X-ray diffraction revealed that the crystalline pattern of water caltrop starch changed from the C_A_-type to the A-type and the relative crystallinity reduced with increasing moisture levels of HMT. Results of ANN-modified water caltrop starch were mostly similar to those of the native one. Moreover, water caltrop starch modified with HMT20 and dual modification contained a pronouncedly higher resistant starch content. These results suggested that HMT, ANN, and dual modification effectively modified the functional properties of water caltrop starch.

## 1. Introduction

Water caltrop (*Trapa Taiwanensis* Nakai) is an aquatic plant belonging to the genus of *Trapa*. It contains various nutrients such as dietary fiber, amino acids, and starch, and has been consumed as traditional food in China and India for years due to the diverse nutritional benefits [1]. Water caltrop contains a large amount of starch, which is a common ingredient for the food industry. Besides, water caltrop starch shows a higher heat stability than lotus rhizome, water chestnuts, and arrowhead starch, and has been getting more attention for food applications in Asia in the past decade [1,2].

Starch is commonly used to modify or improve the texture of food products, like dough [3], yogurt [4], and mayonnaise [5] as a bulking agent, stabilizer, and fat replacer, respectively. However, due to the low shearing resistance, heat resistance, and tendency of retrogradation of native starch, its application is limited [6]. Moreover, the digestibility of starch affects the glucose releasing rate which is a main concern for patients with low glucose tolerance [7]. Hence, resistant starch (RS), which refers to the starch fractions that cannot be digested and absorbed by enzymes in the small intestine, but can be fermented by microorganisms in the large intestine, has been widely researched to fulfill the requirements of controlling the glucose releasing rate and digestibility of starch [8,9,10,11]. For improving the pasting properties and the content of resistant starch, starch modification with physical, chemical, and enzymatic methods were applied [12]. Among them, physical modification is generally considered safe, due to the fact that it involves fewer chemical agents and produces less waste. Heat-moisture treatment (HMT) and annealing (ANN) are common approaches to physically modify starch [13]. Previous studies showed that HMT effectively reduced the swelling power, and viscosity, and increased the gelatinization temperature, retrogradation resistance, and shear resistance of starch [14,15,16]. Furthermore, other research revealed an increment of resistant starch in breadfruit [17], corn, pea, and lentil [18] after HMT. On the other hand, ANN increased the fluidity of the amorphous area of starch due to water absorption, and reversible swelling occurred simultaneously, resulting in improved mobility of the crystalline area [19]. Hence, interactions between molecules were enhanced and produced more even structures which improved the perfection of the crystals. Except for single modification, some studies also combined HMT and ANN for dual modification which showed better heat resistance and a lower tendency to retrograde [20].

In a previous study, water caltrop starch was effectively modified by various thermal treatment/time combination techniques, including microwave, HMT under 30% moisture and pressure heat at 120 °C; ending up with products with different crystallinity, amylose content, resistant starch (RS) content and digestibility [21]. However, no systematic study has been performed to examine the effect of HMT under different moisture levels, ANN, and the combination of both on the properties of water caltrop starch. We hypothesize that more types of physical modification can be tested on water caltrop starch, with a wide range of properties for novel food applications. Therefore, the objective of this study is to investigate the impact of two different hydrothermal treatments (HMT and ANN) and a combination of both on the molecular structure, rheological properties including the pasting property and the final viscosity of starch paste, resistant starch content, and in vitro digestibility of water caltrop starch.

## 2. Results and Discussion

### 2.1. Proximate Compositions

Water caltrop was composed of 0.58% crude lipid, 12.85% crude protein, 1.02% crude fiber, 3.81% ash, and 81.74% nitrogen-free extract (N.F.E.) on a dry basis (d.b.). With the isolation and purification procedure applied, the water caltrop starch obtained was composed of 0.07% crude lipid, 0.59% crude protein, 0.07% ash, and 99.28% nitrogen-free extract (N.F.E.) on a dry basis (d.b.). The high N.F.E. content (above 99%) of the water caltrop starch prepared in this study indicated the fairly high purity of the starch.

### 2.2. Morphology of Starch Granules

**Figure 1:**
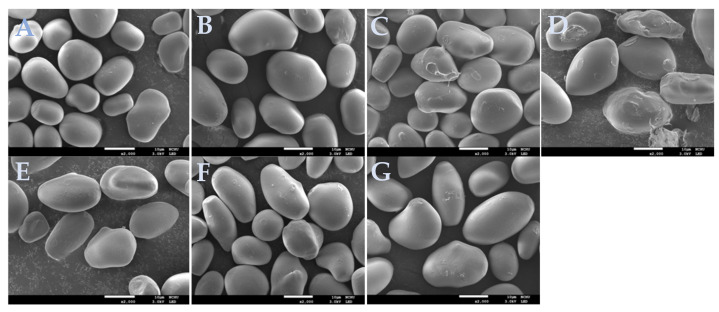
Effect of various hydrothermal treatments on the morphology of water caltrop starch (2000×). (**A**) Native starch; (**B**) HMT20; (**C**) HMT25; (**D**) HMT30; (**E**) ANN; (**F**) HMT20 + ANN; (**G**) ANN + HMT20. HMT indicates heat-moisture treatment. The numbers 20, 25, and 30 indicate the moisture level under HMT. ANN indicates annealing.

Figure 1 presents the micrographs of native and hydrothermally modified water caltrop starch taken from a scanning electron microscope. As shown in Figure 1, native water caltrop starch granules showed irregular shapes but with smooth surfaces. ANN treatment essentially did not impact the surface morphology of starch granules; however, HMT slightly caused dints on the starch granules and they were rougher and irregular, especially in the HMT groups with high moisture levels (25%, 30%). These results were consistent with the findings for HMT modified mango kernel starches and maize starch [22,23], and may be explained by partial gelatinization due to the high temperature applied during HMT. The appearance of dual hydrothermally modified starch was similar to that of HMT20, showing slight dents and partially damaged granules.

### 2.3. Pasting Properties and Iodine Staining Morphology

**Figure 2:**
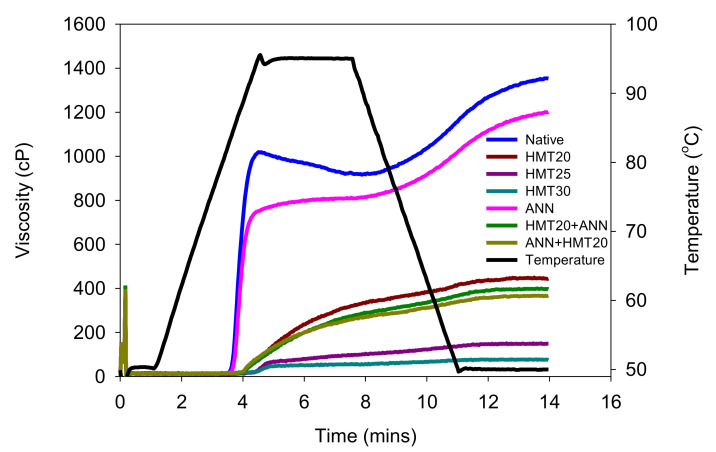
Effect of various hydrothermal treatments on the rapid-visco properties of water caltrop starch. Sample codes refer to Figure 1.

The viscosity profiles during the pasting of water caltrop starch are shown in Figure 2. It was found that the hydrothermal treatments applied in this study may exert molecular changes of water caltrop starch, and modify the rheological properties, including the onset of gelatinization, the peak viscosity, the shear resistance, and the final viscosity of starch pastes. The overall viscosity was decreased significantly by hydrothermal modification (*p* < 0.05) because of the rearrangement of the starch structure that enfeebled the gelatinization and limited the swelling of starch granules. However, it also implied that hydrothermal modification could reduce the effect of shearing force under high temperatures [24,25]. In addition, the pronounced decrement of setback viscosity (*p* < 0.05) indicated that modified starch showed less tendency to retrograde [26]. Moreover, HMT exhibited a more pronounced influence than ANN (*p* < 0.05), and the viscosity decreased gradually with the increased moisture level during HMT (*p* < 0.05), which was possibly due to the shorter chain length of amylopectin and lower swelling power in HMT samples [20].

Table 1 shows the characteristic pasting parameters during rapid-visco analysis (RVA). The peak time and pasting temperature of HMT were both increased pronouncedly compared to the native counterpart (*p* < 0.05), indicating better heat stability, and it became more difficult to gelatinize. This result was in accordance with Ali, et al. [27] who had found similar results on lotus seed starch after HMT. The ANN-modified sample also showed an increasing peak time and pasting temperature because of the improved crystalline perfection and the interaction between molecules that made the structure of starch become more stable and ordered [25,28]. Both dual hydrothermal treatment samples (ANN + HMT20 and HMT20 + ANN) showed a higher pasting temperature and lower viscosity than the single hydrothermal treatment ones (ANN and HMT20) (*p* < 0.05). In addition, ANN + HMT20 showed better heat stability than HMT20 + ANN. The previous study of corn starch modified with the combination of ANN and HMT also showed a lower viscosity than a single modification [20]. These findings revealed that hydrothermal modification of water caltrop starch showed a more stable structure which was more resistant to high temperatures and shearing force. Moreover, the retrogradation of starch paste was also possibly reduced.

**Figure 3:**
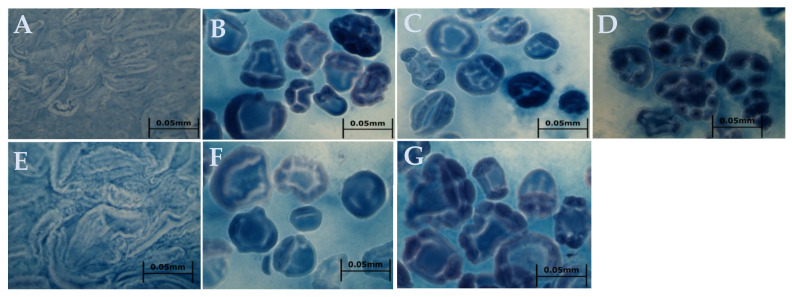
Effect of various hydrothermal treatments on the iodine-staining morphology of water caltrop starch pastes. (**A**) Native starch; (**B**) HMT20; (**C**) HMT25; (**D**) HMT30; (**E**) ANN; (**F**) HMT20 + ANN; (**G**) ANN + HMT20. HMT indicates heat-moisture treatment. The numbers 20, 25, and 30 indicate the moisture level under HMT. ANN indicates annealing.

After pasting in RVA, the appearance of starch paste stained with iodine solution was further examined (Figure 3). It was found that after the heating process in RVA, the native and ANN-modified water caltrop starch granules were essentially burst or destroyed and the starch molecules were leaching out from the granules into the liquid. On the other hand, the margin of starch granules modified by HMT and dual hydrothermal treatments still remained clear, implying that more compact structures were formed by the interaction between starch chains. These results were in line with the pasting properties of hydrothermally modified water caltrop starch, in which HMT and dual modification samples showed pronouncedly decreased viscosity and better resistance to retrogradation (*p* < 0.05). Moreover, there were some light-colored channels inside the starch granules, which also led to an increment of crystal heterogeneity. Some studies about canna, corn, and potato starch also report similar results [29,30,31]. The starch granules of HMT modified samples remained intact granular borderline after heating and implied less susceptibility to shear force at high temperatures. The size of inner channels seemed to be larger with increasing applied moisture levels during HMT, possibly due to higher internal steam pressure generated by HMT, leading to more compact molecular chain arrangement regions which showed higher stability to heat and required a higher temperature for gelatinization. However, these inner channels may make the access of digestive enzymes easier, and affect the digestibility of starch.

### 2.4. Starch Crystalline Pattern and Relative Crystallinity

**Figure 4:**
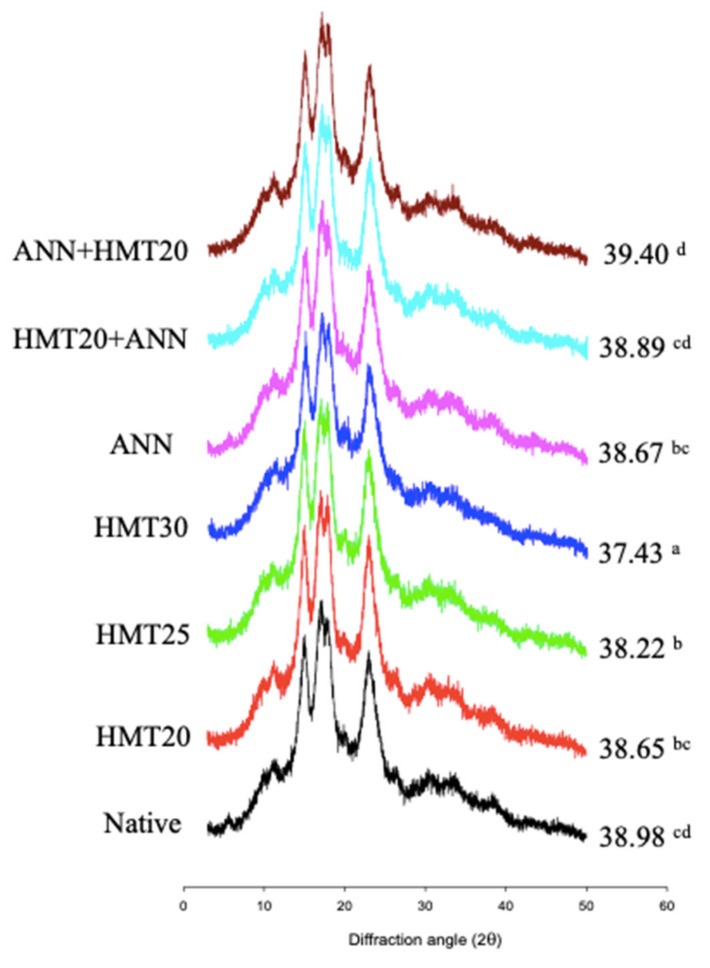
Effect of various hydrothermal treatments on the X-ray diffraction patterns and relative crystallinity of water caltrop starches. Sample codes refer to Figure 1. ^a–d^ Relative crystallinity values with different letters are significantly different (*p* < 0.05).

The results of the X-ray diffraction pattern and relative crystallinity of native and hydrothermally modified water caltrop starch are demonstrated in Figure 4. Native and ANN-modified water caltrop starch exhibited an XRD pattern of the C_A_-type allomorph, characterized by the presence of small intensity peaks at 2θ of 5.6° (typical for B-type), 11° and 20°, strong peaks at 15° and 23° and double peaks at 17° and 18° (typical for A-type). Moreover, the relative crystallinity of the ANN-modified sample showed no significant differences compared to native water caltrop starch. The previous study on potato starch also shows similar results [32]. Though the temperature applied in ANN modification is below the onset temperature of gelatinization, it is higher than the glass transition temperature. In addition, the higher moisture content applied in ANN modification could also have facilitated the mobility of the starch chain. Therefore, though ANN possibly improved the arrangement of the double helix and the crystallite perfection, it would not essentially increase the relative crystallinity [19].

However, for hydrothermally modified samples involved with HMT (both single and dual modification), the peak at 2θ of 5.6° disappeared, implying the crystalline type of HMT-modified starch transformed from C_A_-type to A-type due to HMT modification. The altered crystalline pattern by HMT was also observed in a study by Li et al. [33] who pointed out that HMT made the crystallinity of lily starch change from B-type to A-type. Moreover, reduced relative crystallinity was observed with HMT, and increasing the moisture levels during HMT further lowered the relative crystallinity values. This implied the higher moisture content (25%, 30%) synergized with heat, resulted in the disruption of the original crystal structure, and reduced the relative crystallinity [34]. For dual modification, the relative crystallinity of ANN + HMT20 was slightly higher than ANN, possibly because the starch structure was rearranged during the subsequent HMT to form ordered double helix branched side chain clusters [35].

### 2.5. In Vitro Digestibility

The digestibility of starch is affected by the source, particle size, amylose/amylopectin ratio, crystalline pattern, and amylose–lipid complex [20]. According to the rate of glucose release during starch hydrolysis, Englyst et al. [36] classified starch into three fractions, including rapidly digestible starch (RDS), slowly digestible starch (SDS), and resistant starch (RS). RDS can be converted into glucose within 20 min by digestive enzymes, and it is mainly located in the amorphous region of starch. RDS exists in a large amount in starchy foods after gelatinization, leading to a rapid increase in blood glucose levels after ingestion. SDS can be digested at a slower rate (20 to 120 min) as compared to RDS and it exists in the hard-to-reach parts of the amorphous area and the raw starch of cereals. Both RDS and SDS are considered digestible starches. RS represents the starch fractions that cannot be digested and absorbed by enzymes in the small intestine, but can be fermented by microorganisms in the large intestine. During in vitro digestibility studies of starch, some studies considered starch fractions that cannot be digested within 120 min as RS [21,37]. In this study, we further classified starch into four fractions, they are RDS, SDS, very slow digestive starch (very-SDS) (digested between 120 min to 16 h), and RS (cannot be digested within 16 h). Table 2 shows the results of in vitro digestibility of water caltrop starch. As compared with native starch, single HMT modified water caltrop starch under relatively high moisture levels (HMT25 and HMT30) showed significant increments in rapidly digestible (RDS) and slowly digestible (SDS) starch content, accompanied by a pronounced decrease in very slowly digestible (Very-SDS) and resistant (RS) starch content (*p* < 0.05). This could be explained by the altered crystalline pattern into A-type crystallinity which was composed of shorter A-chains (DP 6–12), mainly located in the crystalline area, and was more likely to be digested by enzymes and so reduced the content of resistant starch [18]. A previous study pointed out that the double helix or relative crystallinity was disrupted by HMT, which loosened the packing of starch, and made it more vulnerable to enzymes that affected the glycosidic bonds [20,38]. Therefore, the destruction of the double helix in the amorphous region during HMT promoted the entry of enzymes into the interaction sites of the amylose molecular chain, and thereby enhanced the enzymatic hydrolysis [39,40]. However, the RDS and SDS contents for HMT20 were lower, and the Very-SDS and RS were higher than the native water caltrop starch. It may be because of the milder condition, coupled with a lesser extent of degradation on water caltrop starch and the formation of interactions between the molecular chains of starch by rearrangement, which reduced the hydrolysis by the enzyme.

As compared with native water caltrop starch, ANN had less impact on the resistant starch content (*p* > 0.05), but slightly decreased the SDS and very-SDS, and increased the RDS significantly (*p* < 0.05). Moreover, for dual hydrothermally modified samples, the RDS increased and SDS decreased slightly, but very-SDS decreased and RS increased pronouncedly (*p* < 0.05). This was possibly due to the fact that the combination of HMT20 and ANN produced more ordered structures between starches that increased the relative crystallinity and enhanced the resistance to the enzymes.

### 2.6. Amylose and Damaged Starch Content

As shown in Table 3, HMT and dual modification slightly increased the amylose content of water caltrop starch. It may be due to the weaker steric hindrance near the α-1, 6 than α-1, 4 glycosidic bond caused by HMT which effectively broke down the α-1, 6 glycosidic bond, destroyed the side chain of amylopectin, and produced the short-chain amylose [41,42]. However, there was no significant difference between HMTs with different moisture contents (*p* > 0.05). ANN had no significant difference compared to native starch, and the trend was consistent with the results of wheat, corn, and potato starch modified by ANN [43].

The squeezing and shearing forces caused by grinding during the processing would produce damaged starch. Starch granules were more likely to absorb water and be affected by enzymes, and in bread processing, an appropriate amount of damaged starch assists the fermentation of bread [44]. HMT increased the damaged starch of water caltrop starch, increasing with the moisture content (*p* < 0.05). Onyango, et al. [45] found similar trends on cassava starch, where the higher moisture content of HMT caused more destruction on starch granules. In the results of SEM, the starch granules of HMT25 and HMT30 were slightly damaged on the surface. In addition, HMT also reduced the relative crystallinity and transformed the crystalline type to A-type which had more branching points and side chains. Therefore, the enzyme hydrolysis was accelerated and increased the damaged starch content, as well as RDS and SDS.

ANN increased the content of damaged starch, and HMT20 + ANN also had a higher damaged starch content than HMT20. It was because the high moisture content of ANN led to an irreversible swell of starch granules, resulting in the leaching out of amylose and facilitated the entry of enzymes for hydrolysis [46]. On the other hand, ANN + HMT20 showed a less damaged starch content and RDS compared to ANN. This may be explained by the promoted intermolecular rearrangement with dual modification, which increased the relative crystallinity and reduced the accessibility of the digestive enzyme.

## 3. Materials and Methods

### 3.1. Materials

Fully matured water caltrops (*Trapa taiwanensis* Nakai) harvested in Taiwan were purchased from Xiaying district of Tainan, Taiwan. The resistant starch assay kit, amylose assay kit, and damage starch assay kit were purchased from Megazyme (Megazyme International Ireland, Co., Wicklow, Ireland). All chemicals and solvents, including sodium hydroxide (Echo Chemical Co., LTD, Miaoli, Taiwan) and 95% ethanol (ChenDing Biological Technology Co., Ltd., Miaoli, Taiwan) were of analytical grade.

### 3.2. Starch Isolation

Water caltrop starch was essentially isolated by using the method of Sun [47]. Briefly, fresh water caltrop kernels were cut into small pieces and homogenized with twice the amount of deionized water in a blender (7012S, Waring Commercial Co. LTD, Stamford, CT, USA). The homogenate was passed through a 100-mesh sieve and the filtered starch milk was allowed to sediment at 4 °C for 12 h. The supernatant was then decanted, and the wet starch was mixed with twice the amount of 0.1% (*w*/*v*) sodium hydroxide solution. The mixture was placed at 4 °C for 12 h, and the brown supernatant was discarded. The wet starch was then mixed with ten times the amount of distilled water by weight to wash out impurities. This washing process was repeated several times until the supernatant was neutral (pH = 7), followed by hot-air drying at 40 °C until the moisture content was less than 10% (d.b.), then starch was ground, sieved through a 100-mesh sieve, and stored at room temperature.

### 3.3. Proximate Composition Analysis

The proximate compositions of water caltrop flour and starch were determined according to AOAC methods [48]. The nitrogen-free extract (N.F.E.) content was calculated as 100 minus the sum of crude lipid, crude protein, crude fiber, and ash contents.

### 3.4. Hydrothermal Treatments

#### 3.4.1. Heat-Moisture Treatment (HMT)

The previous method was essentially applied [28]. Briefly, the moisture levels of starch samples were adjusted to 20%, 25%, and 30% by adding appropriate amounts of deionized water. The mixture was mixed evenly and sealed into tinplate cans (diameter: 8 cm, depth: 5 cm), and tempered at 105 °C for 16 h by using a convective hot-air oven. After modification, the samples were dried at 40 °C until the moisture content was less than 10% (d.b.), then ground with a grinder (RM100, Retsch, Haan, Germany), sieved through a 100-mesh sieve, and stored at room temperature. According to the moisture level applied, HMT-modified samples are denoted hereafter as HMT20, HMT25, and HMT30, respectively.

#### 3.4.2. Annealing Treatment (ANN)

The previous method was essentially applied [28]. The moisture levels of starch samples were adjusted to 70% by adding appropriate amounts of deionized water. The mixture was well stirred, sealed into tinplate cans, and tempered at 50 °C for 24 h by using a convective hot-air oven. After modification, the samples were dried at 40 °C until the moisture content was less than 10% (d.b.), then ground, sieved as described in Section 3.4.1, and stored at room temperature. The annealed starch sample is hereafter referred to as ANN.

#### 3.4.3. Dual Hydrothermal Treatment

Dual hydrothermal modification of water caltrop starch was performed by applying HMT20 and ANN treatment in different sequences. For the HMT20 + ANN sample, water caltrop starch was subjected to HMT20 treatment (as described in Section 3.4.1), followed by being subjected to ANN treatment (as described in Section 3.4.2). In contrast, for the ANN + HMT20 sample, water caltrop starch was subjected to ANN treatment (as described in Section 3.4.2) followed by being subjected to heat-moisture treatment at a 20% moisture level (as described in Section 3.4.1).

### 3.5. Morphological Observation of Starch Granules

Native and hydrothermally modified water caltrop starch were transferred with double-sided carbon adhesive tapes and then coated with platinum films using a metal ion coater (JEC-3000FC, JEOL, Auto Fine Coater, Tokyo, Japan). The observation was performed with a scanning electron microscope (JSM-7800F, JEOL, Tokyo, Japan) under a 3.0 kV accelerating voltage.

### 3.6. Pasting Properties and Iodine Staining Observation of Starch Paste

The pasting properties of native and hydrothermally modified water caltrop starch were evaluated by using the method of [47] with a rapid-visco analyzer (RVA-Ezi, Newport Scientific Pty Limited, Warriewood NSW, Australia). Briefly, a 6% starch suspension was prepared by mixing the starch samples with the proper amount of deionized water in an aluminum cylinder. The following conditions were used for analysis: holding at 50 °C for 1 min, heating to 95 °C at a rate of 12.86 °C/min, holding at 95 °C for 3 min, cooling to 50 °C at a rate of 12.86 °C/min, and holding at 50 °C for 3 min. An initial mixing speed of 960 rpm was applied to disperse the starch sample for the first 10 s and a constant mixing speed of 160 rpm was used for the rest of the experiment.

The morphology of starch granules after being gelatinized by RVA was observed by staining aliquots of samples on the glass slides with iodine solution (0.2% I_2_/KI). The observation with a light microscope (BX41, Olympus, Tokyo, Japan) was recorded by a digital camera (E330, Olympus, Tokyo, Japan) at a 400× magnification.

### 3.7. Starch Crystalline Pattern and Relative Crystallinity

Native and hydrothermally modified water caltrop starch samples were equilibrated in a glass desiccator filled with saturated sodium chloride solution (RH = 75%) for one week, in order to stabilize the starch granule structure [49]. Measurement was done with an X-ray diffractometer (X’Pert Pro MRD, PANalytical, Almelo, The Netherlands) equipped with copper radiation (Cu K_α_, λ = 0.15418 nm). The scanning angle 2θ was applied and scanned from 3° to 50° under a voltage of 45 kV and a current of 40 mA with a scan step of 0.02°. PeakFit software (v4.00, 1995, Jandel Scientific Software, AISN Software Inc., Erkrath, Germany) was utilized for calculating the relative crystallinity with the following equation,

(1) $$Xc_{\;}=\;\frac{I_{c}}{I_{c}+I_{a}}$$

where X_c_ is the relative crystallinity (%) of the samples; I_c_: the integration of diffraction intensity at the crystalline part; I_a_: the integration of diffraction intensity at the amorphous part.

### 3.8. In Vitro Digestibility

In vitro digestibility of native and hydrothermally modified water caltrop starch samples were determined by the Megazyme resistant starch kit (K-RSTAR, Megazyme International Ireland, Co., Wicklow, Ireland) according to the previous method with modifications [36,50]. The rapidly digestible starch (RDS), slowly digestible starch (SDS), very slowly digestible starch (Very-SDS), and resistant starch (RS) were calculated with the following equations:

(2) $$\mathrm{RDS}\;(\%)\;=\;\frac{G_{20\;\mathrm{mins}}\times 0.9}{\;\mathrm{TS}}$$

(3) $$\mathrm{SDS}\;(\%)\;=\;\frac{(G_{120\;\mathrm{mins}}-G_{20\;\mathrm{mins}})\times 0.9}{\mathrm{TS}}$$

(4) $$\mathrm{Very}-\mathrm{SDS}\;(\%)\;=\;\frac{(G_{16\;\mathrm{hrs}}-G_{120\;\mathrm{mins}})\times 0.9}{\mathrm{TS}}$$

(5) $$\mathrm{RS}\;(\%)\;=\;\frac{\mathrm{TS}-\left(\mathrm{Very}\;\mathrm{SDS}+\mathrm{SDS}+\mathrm{RDS}\right)}{\mathrm{TS}}$$

where RDS is rapidly digestible starch, SDS is slowly digestible starch, Very-SDS is very-slowly digestible starch, RS is resistant starch, G_time_ is the glucose content released at the time, and TS is the total starch content of the samples.

### 3.9. Amylose and Damaged Starch Content

Amylose and damage starch content of native and hydrothermally modified water caltrop starch were determined by using the Megazyme amylose kit (K-AMYL) and damage starch kit (K-SDAM) according to the respective analysis procedures provided by the manufacturer.

### 3.10. Statistical Analysis

Analysis of variance was performed using SPSS 19 (IBM Corp., Armonk, NY, USA, 2010) and the data were expressed as mean ± standard deviation (*n* = 3). The post-hoc analysis of Duncan’s multiple range test was applied at a confidence interval of 95% (*p* < 0.05).

## 4. Conclusions

After being treated by HMT, slightly dented or damaged regions on the surface of water caltrop starch granules were noticed, though the geometry of starch granules essentially remained intact. The high energy provided by HMT may disrupt the α-1,6 glycosidic bonds, resulting in an increased amylose content and lowered relative crystallinity. Moreover, HMT may promote molecular rearrangement, leading to modifying the crystalline type of water caltrop starch from C_A_-type to A-type. The hydrothermal tempering condition of ANN used in this study was much milder than HMT, and results of ANN-modified water caltrop starch were mostly similar to those of the native one, though ANN slightly improved the thermal stability of water caltrop starch. Speaking overall, the hydrothermally modified water caltrop starch involved with HMT generally exhibited much lower viscosity and higher shear resistance at high temperatures, implying suitability for canned foods. Moreover, water caltrop starch modified with HMT20 and dual modification (HMT20 + ANN and ANN + HMT20) contained a pronouncedly higher resistant starch content and showed great potential in regulating blood sugar. These hydrothermally modified water caltrop starches can be further explored as functional ingredients to be added to daily foods or other starch products for better nutrition or improved thermal/shear stability in food processing. Nevertheless, the in vitro digestibility may not completely reflect the exact digestion in vivo, and more research is needed to have a better understanding of the mechanism in vivo.

## Figures and Tables

**Table 1:** Effect of various hydrothermal treatments on the pasting parameters of water caltrop starch ^1,2^.

Samples	Pasting Temperature (°C)	Peak Time (min)	Peak Viscosity	Holding Strength	Final Viscosity	Setback
			(cP)			
Native	82.93 ± 0.25 ^a^	4.32 ± 0.04 ^a^	976.00 ± 4.36 ^f^	919.67 ± 7.37 ^g^	1366.67 ± 16.50 ^g^	447.00 ± 10.54 ^f^
HMT20	88.30 ± 0.33 ^d^	4.60 ± 0.03 ^b^	88.00 ± 3.61 ^d^	314.33 ± 5.13 ^e^	438.67 ± 5.03 ^e^	124.33 ± 3.51 ^d^
HMT25	94.40 ± 0.22 ^e^	4.93 ± 0.04 ^c^	64.33 ± 0.58 ^b^	95.00 ± 1.73 ^b^	145.67 ± 5.77 ^b^	50.67 ± 4.04 ^b^
HMT30	95.25 ± 0.40 ^f^	4.98 ± 0.13 ^c^	45.33 ± 1.53 ^a^	53.33 ± 0.58 ^a^	74.67 ± 1.16 ^a^	21.33 ± 0.58 ^a^
ANN	84.02 ± 0.08 ^b^	4.27 ± 0.00 ^a^	715.67 ± 4.73 ^e^	802.00 ± 5.20 ^f^	1196.67 ± 4.04 ^f^	394.67 ± 2.08 ^e^
HMT20 + ANN	87.67 ± 0.45 ^c^	4.57 ± 0.04 ^b^	77.67 ± 1.16 ^c^	268.33 ± 9.87 ^d^	392.67 ± 9.45 ^d^	124.33 ± 1.16 ^d^
ANN + HMT20	88.45 ± 0.26 ^d^	4.50 ± 0.00 ^b^	85.00 ± 3.61 ^d^	257.33 ± 2.89 ^c^	365.00 ± 2.65 ^c^	107.67 ± 0.58 ^c^

^1^ Sample codes refer to Figure 1. ^2^ Each data was expressed as the mean ± standard deviation (*n* = 3). Means with different superscript letters in the same column are significantly different (*p* < 0.05).

**Table 2:** Effect of various hydrothermal treatments on the in vitro digestibility of water caltrop starch ^1,2^.

Samples	RDS (20 min)	SDS (20–120 min)	Very-SDS (120 min–16 h)	RS (>16 h)
	(%, d.b.)			
Native	5.69 ± 0.16 ^b^	9.02 ± 0.22 ^b^	40.29 ± 0.43 ^c^	37.18 ± 0.55 ^c^
HMT20	4.03 ± 0.09 ^a^	7.32 ± 0.61 ^a^	41.76 ± 3.05 ^c^	40.19 ± 0.63 ^d^
HMT25	8.40 ± 0.12 ^f^	10.72 ± 0.13 ^c^	38.77 ± 1.69 ^bc^	32.44 ± 0.05 ^b^
HMT30	26.29 ± 0.05 ^g^	12.37 ± 1.47 ^d^	36.09 ± 1.72 ^b^	17.01 ± 0.08 ^a^
ANN	7.71 ± 0.12 ^e^	8.24 ± 0.29 ^ab^	38.43 ± 1.48 ^bc^	37.74 ± 1.30 ^c^
HMT20 + ANN	6.62 ± 0.05 ^d^	6.85 ± 1.52 ^a^	32.09 ± 1.88 ^a^	43.90 ± 1.64 ^e^
ANN + HMT20	5.87 ± 0.03 ^c^	7.28 ± 0.08 ^a^	35.36 ± 2.14 ^ab^	43.94 ± 0.84 ^e^

^1^ Sample codes refer to Figure 1. ^2^ Each datum was expressed as the mean ± standard deviation (*n* = 3). Means in the same column with different superscript letters are significantly different (*p* < 0.05).

**Table 3:** Effect of various hydrothermal treatments on the amylose and damaged starch content of water caltrop starch ^1,2^.

Samples	Amylose	Damage Starch
	(%, d.b.)	
Native	23.29 ± 0.90 ^ab^	1.38 ± 0.01 ^a^
HMT20	24.20 ± 0.83 ^bc^	1.47 ± 0.03 ^a^
HMT25	24.67 ± 1.14 ^cd^	1.87 ± 0.06 ^b^
HMT30	25.05 ± 0.71 ^cd^	8.06 ± 0.22 ^e^
ANN	22.91 ± 0.17 ^a^	2.71 ± 0.04 ^c^
HMT20 + ANN	25.78 ± 0.28 ^de^	3.41 ± 0.03 ^d^
ANN + HMT20	26.41 ± 0.09 ^e^	1.71 ± 0.03 ^b^

^1^ Sample codes refer to Figure 1. ^2^ Each datum was expressed as the mean ± standard deviation (*n* = 3). Data in the same column with different superscript letters are significantly different (*p* < 0.05).

## Data Availability

The data presented in this study are available on request from the corresponding author. The data are not publicly available due to ethical restrictions and intellectual property issues.

## Sample Availability

The samples are not publicly available due to the funding limitation.

## References

[1] Zhu F. (2016). Chemical composition, health effects, and uses of water caltrop. Trends Food Sci. Technol..

[2] Wang J., Liu T., Bian X., Hua Z., Chen G., Wu X. (2021). Structural characterization and physicochemical properties of starch from four aquatic vegetable varieties in China. Int. J. Biol. Macromol..

[3] Durmus Y., Anil M., Simsek S. (2021). Effects of hazelnut skin, cross-linked starch, and oxidized starch on wheat flour and dough quality. J. Food Process. Preserv..

[4] Altemimi A.B. (2018). Extraction and optimization of potato starch and its application as a stabilizer in yogurt manufacturing. Foods.

[5] Heydari A., Razavi S.M.A., Farahnaky A. (2021). Effect of high pressure-treated wheat starch as a fat replacer on the physical and rheological properties of reduced-fat O/W emulsions. Innov. Food Sci. Emerg. Technol..

[6] Kaur L., Singh J., Caballero B., Finglas P.M., Toldrá F. (2016). Starch: Modified starches. Encyclopedia of Food and Health.

[7] Sandberg J.C., Björck I.M., Nilsson A.C. (2017). Effects of whole grain rye, with and without resistant starch type 2 supplementation, on glucose tolerance, gut hormones, inflammation and appetite regulation in an 11–14.5 hour perspective; a randomized controlled study in healthy subjects. Nutr. J..

[8] Remya R., Jyothi A.N., Sreekumar J. (2018). Effect of chemical modification with citric acid on the physicochemical properties and resistant starch formation in different starches. Carbohydr. Polym..

[9] Su C., Zhao K., Zhang B., Liu Y., Jing L., Wu H., Gou M., Jiang H., Zhang G., Li W. (2020). The molecular mechanism for morphological, crystal, physicochemical and digestible property modification of wheat starch after repeated versus continuous heat-moisture treatment. LWT.

[10] Raigond P., Ezekiel R., Raigond B. (2015). Resistant starch in food: A review. J. Sci. Food Agric..

[11] Jiang F., Du C., Jiang W., Wang L., Du S.-k. (2020). The preparation, formation, fermentability, and applications of resistant starch. Int. J. Biol. Macromol..

[12] Kaur B., Ariffin F., Bhat R., Karim A.A. (2012). Progress in starch modification in the last decade. Food Hydrocoll..

[13] Schafranski K., Ito V.C., Lacerda L.G. (2021). Impacts and potential applications: A review of the modification of starches by heat-moisture treatment (HMT). Food Hydrocoll..

[14] Kaur M., Singh S. (2019). Influence of heat-moisture treatment (HMT) on physicochemical and functional properties of starches from different Indian oat (*Avena sativa* L.) cultivars. Int. J. Biol. Macromol..

[15] Molavi H., Razavi S.M.A., Farhoosh R. (2018). Impact of hydrothermal modifications on the physicochemical, morphology, crystallinity, pasting and thermal properties of acorn starch. Food Chem..

[16] Yassaroh Y., Woortman A.J.J., Loos K. (2019). A new way to improve physicochemical properties of potato starch. Carbohydr. Polym..

[17] Tan X., Li X., Chen L., Xie F., Li L., Huang J. (2017). Effect of heat-moisture treatment on multi-scale structures and physicochemical properties of breadfruit starch. Carbohydr. Polym..

[18] Chung H.-J., Liu Q., Hoover R. (2009). Impact of annealing and heat-moisture treatment on rapidly digestible, slowly digestible and resistant starch levels in native and gelatinized corn, pea and lentil starches. Carbohydr. Polym..

[19] Jayakody L., Hoover R. (2008). Effect of annealing on the molecular structure and physicochemical properties of starches from different botanical origins—A review. Carbohydr. Polym..

[20] Chung H.-J., Hoover R., Liu Q. (2009). The impact of single and dual hydrothermal modifications on the molecular structure and physicochemical properties of normal corn starch. Int. J. Biol. Macromol..

[21] Wei H.-X., Liang B.-D., Chai Y.-R., Xue L.-P., Wang X.-Q., Yin X.-M. (2020). Effect of different heat treatments on physicochemical properties and structural and digestibility of water caltrop starch. Starch Stärke.

[22] Bharti I., Singh S., Saxena D.C. (2019). Exploring the influence of heat moisture treatment on physicochemical, pasting, structural and morphological properties of mango kernel starches from Indian cultivars. LWT Food Sci. Technol..

[23] Liu H., Lv M., Wang L., Li Y., Fan H., Wang M. (2016). Comparative study: How annealing and heat-moisture treatment affect the digestibility, textural, and physicochemical properties of maize starch. Starch Stärke.

[24] Yadav B.S., Guleria P., Yadav R.B. (2013). Hydrothermal modification of Indian water chestnut starch: Influence of heat-moisture treatment and annealing on the physicochemical, gelatinization and pasting characteristics. LWT Food Sci. Technol..

[25] da Rosa Zavareze E., Dias A.R.G. (2011). Impact of heat-moisture treatment and annealing in starches: A review. Carbohydr. Polym..

[26] Singh H., Chang Y.H., Lin J.-H., Singh N., Singh N. (2011). Influence of heat–moisture treatment and annealing on functional properties of sorghum starch. Food Res. Int..

[27] Ali N.A., Dash K.K., Routray W. (2020). Physicochemical characterization of modified lotus seed starch obtained through acid and heat moisture treatment. Food Chem..

[28] Chung H.J., Liu Q., Hoover R. (2010). Effect of single and dual hydrothermal treatments on the crystalline structure, thermal properties, and nutritional fractions of pea, lentil, and navy bean starches. Food Res. Int..

[29] Kawabata A., Takase N., Miyoshi E., Sawayama S., Kimura T., Kudo K. (1994). Microscopic observation and X-ray diffractometry of heat/moisture-treated starch granules. Starch Stärke.

[30] Vermeylen R., Goderis B., Delcour J.A. (2006). An X-ray study of hydrothermally treated potato starch. Carbohydr. Polym..

[31] Watcharatewinkul Y., Puttanlek C., Rungsardthong V., Uttapap D. (2009). Pasting properties of a heat-moisture treated canna starch in relation to its structural characteristics. Carbohydr. Polym..

[32] Stute R. (1992). Hydrothermal modification of starches: The difference between annealing and heat/moisture-treatment. Starch Stärke.

[33] Li H., Wang R., Liu J., Zhang Q., Li G., Shan Y., Ding S. (2020). Effects of heat-moisture and acid treatments on the structural, physicochemical, and in vitro digestibility properties of lily starch. Int. J. Biol. Macromol..

[34] Bet C.D., de Oliveira C.S., Colman T.A.D., Marinho M.T., Lacerda L.G., Ramos A.P., Schnitzler E. (2018). Organic amaranth starch: A study of its technological properties after heat-moisture treatment. Food Chem..

[35] Li S., Ward R., Gao Q. (2011). Effect of heat-moisture treatment on the formation and physicochemical properties of resistant starch from mung bean (Phaseolus radiatus) starch. Food Hydrocolloids.

[36] Englyst H.N., Kingman S., Cummings J. (1992). Classification and measurement of nutritionally important starch fractions. Eur. J. Clin. Nutr..

[37] Van Hung P., Binh V.T., Nhi P.H.Y., Phi N.T.L. (2020). Effect of heat-moisture treatment of unpolished red rice on its starch properties and in vitro and in vivo digestibility. Int. J. Biol. Macromol..

[38] Chen Y., Yang Q., Xu X., Qi L., Dong Z., Luo Z., Lu X., Peng X. (2017). Structural changes of waxy and normal maize starches modified by heat moisture treatment and their relationship with starch digestibility. Carbohydr. Polym..

[39] Chi H.-Y. (2018). Effect of Heat-Moisture Treatments on the Digestibility and Physicochemical Properties of Tainung No.57 and Tainung No.66 Sweet Potato Starches. Master’s Thesis.

[40] Gunaratne A., Hoover R. (2002). Effect of heat–moisture treatment on the structure and physicochemical properties of tuber and root starches. Carbohydr. Polym..

[41] Liu K., Zhang B., Chen L., Li X., Zheng B. (2019). Hierarchical structure and physicochemical properties of highland barley starch following heat moisture treatment. Food Chem..

[42] Wang H., Zhang B., Chen L., Li X. (2016). Understanding the structure and digestibility of heat-moisture treated starch. Int. J. Biol. Macromol..

[43] Kohyama K., Sasaki T. (2006). Differential scanning calorimetry and a model calculation of starches annealed at 20 and 50 C. Carbohydr. Polym..

[44] Wang S., Yu J., Xin Q., Wang S., Copeland L. (2017). Effects of starch damage and yeast fermentation on acrylamide formation in bread. Food Control.

[45] Onyango C., Mewa E.A., Mutahi A.W., Okoth M.W. (2013). Effect of heat-moisture-treated cassava starch and amaranth malt on the quality of sorghum-cassava-amaranth bread. Afr. J. Food Sci..

[46] Wang S., Jin F., Yu J. (2013). Pea starch annealing: New insights. Food Bioprocess Technol..

[47] Sun Y.-Y. (2009). Effects of Different Sugar/Salt Solutions on the Physical Properties of Purple Jade Sweet Potato Starch. Master’s Thesis.

[48] Latimer G. (2021). Official Methods of Analysis of AOAC International.

[49] Yeh Y. (2019). Effect of Single and Dual Hydrothermal Treatments on the Physicochemical Properties and Digestibility of Lotus Rhizome Starches. Master’s Thesis.

[50] Boonna S., Tongta S. (2018). Structural transformation of crystallized debranched cassava starch during dual hydrothermal treatment in relation to enzyme digestibility. Carbohydr. Polym..

